# Sulfated Hyaluronan in Dermatology: What’s New? Overview of Evidence in Specific Dermatological Diseases

**DOI:** 10.3390/pharmaceutics17121600

**Published:** 2025-12-12

**Authors:** Giovanni Pellacani, Simone Michelini, Federica Trovato, Alessandra Rallo, Giuseppe Gemma, Camilla Chello, Mauro Pavan, Cristian Guarise, Alberto Giuseppe Passi

**Affiliations:** 1 Dermatology Unit, Department of Clinical Internal, Anesthesiological and Cardiovascular Science, Sapienza University, 00185 Rome, Italysimone.michelini@uniroma1.it (S.M.);; 2 Department of Regenerative and Immunological Dermatology, Istituto Dermopatico dell’Immacolata IDI-IRCCS, 00167 Rome, Italy; 3 Fidia Farmaceutici S.p.A., 35031 Abano Terme, Italy; 4 Department of Medicine and Surgery, University of Insubria, 21100 Varese, Italy

**Keywords:** hyaluronic acid, sulfated hyaluronan, glycosaminoglycans, oxidative stress, skin barrier, topical delivery, cosmeceuticals, dermatology

## Abstract

**Background/Objectives:** Sulfated hyaluronic acid (sHA) is a chemically modified derivative of native hyaluronic acid (HA), characterized by enhanced physicochemical stability and increased biological activity. Glycosaminoglycans (GAGs), including HA, are key regulators of skin structure, hydration, and immune homeostasis. This review aims to critically summarize current knowledge on the structural and functional properties of GAGs—particularly HA and its sulfated forms—and to explore their potential dermatological applications in skin aging and inflammatory diseases such as atopic dermatitis, psoriasis, and acne. **Methods:** A narrative literature review was conducted using PubMed and Scopus databases up to June 2025, including experimental, preclinical, and clinical studies investigating the biological effects, mechanisms of action, and dermatological uses of sHA compared with native HA and other HA derivatives. **Results:** Compared with HA, sHA demonstrates increased enzymatic resistance, higher charge density, and improved water-binding and antioxidant capacity. These properties contribute to the restoration of skin barrier function, modulation of oxidative stress and inflammation, and promotion of extracellular matrix remodeling. Preclinical evidence supports the efficacy of sHA in reducing dryness, irritation, and inflammatory responses in atopic dermatitis, psoriasis, and acne. Preliminary findings also suggest potential benefits in wound healing and skin barrier repair. **Conclusions:** sHA represents a promising multifunctional molecule in dermatology and cosmetology, capable of reducing inflammation and supporting tissue regeneration. However, current evidence remains limited to preliminary studies. Future controlled clinical trials are required to confirm efficacy, optimize formulations, and establish standardized treatment protocols.

## 1. Introduction

Hyaluronic acid (HA) is a linear, non-sulfated glycosaminoglycan composed of repeating disaccharide units of N-acetylglucosamine and glucuronic acid. It is an essential component of the extracellular matrix (ECM), contributing to tissue hydration, elasticity, and cellular communication. In the skin, HA plays a pivotal role in maintaining epidermal barrier integrity and modulating inflammation and wound healing. However, native HA is characterized by a relatively short half-life and is rapidly degraded by hyaluronidases and reactive oxygen species, limiting its long-term efficacy in topical and injectable formulations [1,2].

To overcome these limitations, various HA derivatives have been developed through chemical modifications aimed at enhancing stability and biological performance. These include cross-linked HA (used in dermal fillers for prolonged residence time), acetylated HA (with improved resistance to enzymatic degradation), carboxymethylated HA (offering better mucoadhesion), and benzyl ester HA (used in tissue engineering and wound repair) [3,4,5,6,7]. Among these, sulfated hyaluronic acid (sHA) has recently attracted considerable attention due to its structural similarity to naturally occurring sulfated glycosaminoglycans (GAGs) such as heparan sulfate and chondroitin sulfate [7].

The introduction of sulfate groups confers increased negative charge density, enhancing water-binding capacity, antioxidant potential, and electrostatic interactions with growth factors, cytokines, and ECM proteins. These features suggest that sHA may act not only as a humectant but also as a bioactive molecule capable of modulating inflammation, oxidative stress, and tissue regeneration [7,8,9,10,11,12].

Despite these promising properties, evidence on the dermatological applications of sHA remains scattered and largely preclinical. A comprehensive synthesis is therefore needed to clarify its mechanisms of action, therapeutic potential, and current research gaps.

This review aims to critically evaluate the structural, biological, and functional characteristics of sulfated hyaluronic acid (sHA), with emphasis on its dermatological relevance and emerging applications in inflammatory and barrier-related skin diseases.

Sulfation of HA markedly reduces its susceptibility to hyaluronidase degradation and increases its negative charge density, thereby strengthening electrostatic interactions with binding proteins and growth factors such as VEGF, FGF, and TGF-β. These biochemical properties provide a strong rationale for the use of sHA in topical formulations and tissue repair matrices [12,13].

## 2. Hyaluronic Acid: Biosynthesis and Role in Normal and Aged Skin

Hyaluronic acid (HA) is a non-sulfated GAG and a major structural component of the extracellular matrix (ECM) [14] In its native form, HA exists mainly as a high-molecular-weight polymer (HMW-HA), composed of alternating units of D-glucuronic acid and N-acetylglucosamine linked by β-1,3 and β-1,4 glycosidic bonds. Under physiological or oxidative conditions, HA can be enzymatically cleaved into low-molecular-weight fragments (LMW-HA) with distinct biological functions [15] (Table 1).

Unlike other GAGs synthesized in the Golgi apparatus, HA is produced at the plasma membrane by three hyaluronan synthase (HAS) isoenzymes—HAS1, HAS2, and HAS3—which extrude HA directly into the extracellular space. This enables the generation of large polymers without compromising cellular integrity [8,12].

HA plays a critical role in maintaining ECM structure, hydration, and viscoelasticity. Through its interaction with cell-surface receptors such as CD44 and RHAMM, HA regulates keratinocyte proliferation, fibroblast migration, and inflammatory signaling. Its physicochemical and biological behavior are primarily determined by its molecular weight, which influences diffusion, receptor binding, and enzymatic degradation. The polymer’s size is directly related to its chemical stability and tissue diffusion capacity: in particular, HA chains within the 50–500 kDa range display superior structural stability and higher permeability within the cutaneous microenvironment compared to smaller oligosaccharides, as first described by Baggenstoss et al. [7,8,16,17].

During intrinsic aging and photoaging, HA undergoes quantitative and qualitative alterations. Epidermal HA expression markedly decreases, leading to dryness and loss of elasticity, whereas dermal HA becomes more fragmented due to reactive oxygen species (ROS) and hyaluronidase activity. These changes contribute to ECM disorganization and impaired barrier function [18,19].

In wound healing, low- to medium-molecular-weight HA (50–500 kDa) supports re-epithelialization by promoting keratinocyte migration and collagen remodeling, while HMW-HA (>1000 kDa) primarily provides structural integrity and anti-inflammatory protection. However, both forms are rapidly degraded in vivo, limiting their long-term efficacy in topical or injectable formulations [20].

To overcome this limitation, chemical modification strategies have been developed to enhance HA’s stability, resistance to enzymatic degradation, and biological activity. Among them, sulfation represents a particularly promising approach, as it increases HA’s negative charge density and affinity for bioactive molecules, mimicking the structural and functional properties of naturally sulfated GAGs such as heparan sulfate and chondroitin sulfate [21].

## 3. Sulfated GAGs: Biosynthesis and Role in Normal and Aged Skin

**Figure 1:**
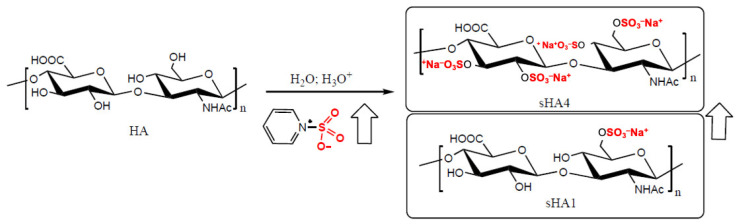
Example of the synthesis of sulfated HA (sHA) starting from HA and pyridine sulfur trioxide (PyrSO_3_). By increasing the amount of PyrSO_3_, the degree of sulfation increases from 1 to 4. Experimentally, for steric and electrostatic reasons, the maximum degree of sulfation achievable is hardly above 3 (sHA3). The arrows indicate the progressive increase in the degree of sulfation.

Sulfated GAGs, including chondroitin sulfate (CS), dermatan sulfate (DS), heparan sulfate (HS), and keratan sulfate (KS) (Figure 1), are synthesized through a tightly regulated multi-step process that primarily occurs in the Golgi apparatus. Biosynthesis begins with the formation of a tetrasaccharide linker on specific serine residues of core proteins, serving as the primer for GAG chain elongation [22]. Sequential addition of alternating hexosamine and uronic acid residues by specific glycosyltransferases leads to polymer formation. Subsequent sulfation—catalyzed by a family of sulfotransferases using 3′-phosphoadenosine-5′-phosphosulfate (PAPS) as the universal sulfate donor—introduces sulfate groups at defined positions along the sugar backbone. The degree and pattern of sulfation determine the binding affinity of sGAGs for growth factors, cytokines, and ECM proteins, conferring distinct biological functions [23,24,25,26].

Structurally, sGAGs are covalently linked to core proteins to form proteoglycans, integral components of the ECM scaffold. These complexes interact with collagen and elastin fibers, providing mechanical stability, elasticity, and resilience to the dermal matrix. Their extended, negatively charged polysaccharide chains generate a hydrated, gel-like microenvironment that maintains turgor and facilitates the diffusion of nutrients and signaling molecules (Table 2). The high density of sulfate and carboxyl groups enables electrostatic retention of water and growth factors, essential for maintaining skin elasticity and barrier integrity [25,27].

With chronological aging, total sGAG content decreases, whereas the relative composition of individual species (e.g., CS, DS, HS) becomes altered [28]. These changes reduce the ECM’s ability to bind water and sequester morphogens, thereby compromising dermal structure and repair capacity. Furthermore, alterations in sulfation pattern and chain length affect electrostatic interactions with key regulatory molecules, impairing pathways involved in wound healing, angiogenesis, and collagen synthesis [29]. In aged skin, proteoglycans such as decorin exhibit shortened GAG chains and diminished capacity to organize collagen fibrils, leading to ECM disorganization, wrinkle formation, and loss of elasticity [30].

**Table 2:** Structure of the five main sulfated GAGs: Heparan sulfate (HS) [31]; Heparin; Keratan sulfate (KS) [32]; Chondroitin sulfate (CS) [33] and Dermatan sulfate (DS) [34], the non-sulfated GAG: Hyaluronic acid (HA) [35] and semisynthetic sulfated HA (sHA) [36,37] at different degree of sulfation (DS = average number of -SO_3_Na groups per disaccharide repeating unit). The figure shows for each polymer: composition; MW and sulfation degree per dimer vs. possible sulfated sites.

Polymer	Sugar 1	Sugar 2	MW (kDa)	DS (mol)	R1	R2,2,4	Sulphation Ratio for Dimer	Reference
HS	GlcA/IdoA	GlcN	~30	1–2	Ac	-SO_3_Na/-H	1/3~2/3	[31]
Heparin	GlA/Ido	GlcN	~15	2–3	-SO_3_Na	-SO_3_Na/-H	2/4~3/4	[31]
KS	Gal	GlcNAc	36–41	1–2	-SO_3_Na/-H	-SO_3_Na/-H	1/2~2/2; SO3Na; R1 ≠ R2 ≠ 3,4	[32]
CS	GlcA	GalNAc	50–100	1–2	-SO_3_Na/-H	-SO_3_Na/-H	1/4~2/4; -SO_3_Na; R1,2 > R3,4	[33]
DS	IdoA	GalNAc	30–92	1–2	-SO_3_Na/-H	-SO_3_Na/-H	1/3–2/3	[34]
HA	GlcA	GlcNAc	200–8000	0	-H	-H	0	[35]
sHA1	GlcA	GlcNAc	15–43	0.5–1	-SO_3_Na	-H	~1/4	[36]
sHA2	GlcA	GlcNAc	~1–20	~1/2.2	-SO_3_Na	-SO_3_Sa	~2/4	[36]
sHA3	SHA	GlcNAc	15–43	2–3	-SO_3_Na	-SO_3_Sa	~3/4	[36]

From a dermatological perspective, these findings underscore the critical role of sulfation in determining the biophysical and biological functions of GAGs. The introduction of sulfate groups not only increases molecular stability and charge density but also enhances the capacity of GAGs to bind and regulate signaling molecules. By mimicking these natural features, sulfated hyaluronic acid (sHA) aims to restore some of the lost functions of native HA, combining hydration, antioxidant protection, and biochemical signaling modulation, properties that justify its exploration as a next-generation bioactive polymer in dermatological formulations [8,12,37,38].

## 4. Sulfated Hyaluronan: Chemical Properties

Figure 1 summarizes the chemical structure of the main sulfated glycosaminoglycans (sGAGs) and of the non-sulfated glycosaminoglycan hyaluronic acid (HA). sGAGs generally exhibit low molecular weight (<0.1 MDa) and moderate sulfation degree (DS 1–2); however, variations in sugar composition and sulfation patterns confer distinct physicochemical and biological functions. Among them, heparin shares structural similarity with heparan sulfate but possesses a higher sulfation degree (DS 2–3), which accounts for its potent anticoagulant activity [39].

In contrast, HA is the only non-sulfated GAG and is characterized by a much higher molecular weight (0.2–8 MDa), responsible for its exceptional lubricating and viscoelastic properties [40].

Historically, both chondroitin sulfate (CS) and HA were extracted from animal tissues through time-consuming and contamination-prone procedures. Currently, HA—and to a lesser extent CS—is industrially produced by microbial fermentation, ensuring high purity and reproducibility. Beyond its unmodified form, HA can undergo several chemical modifications to enhance mechanical performance, bioactivity, or in vivo stability [41].

With the aim of mimicking the structural and functional properties of natural sulfated GAGs, sulfated hyaluronic acid (sHA) has been developed as a derivative of HA through the sulfation of one or more hydroxyl groups of the repeating disaccharide unit. This chemical modification substantially increases the negative charge density of the polymer and improves its water solubility. According to published methods, sHA can be synthesized by reacting the tetrabutylammonium salt of HA with a sulfating agent (e.g., SO_3_–pyridine complex) in an acidic organic environment [42] or directly from the sodium salt of HA in acidic aqueous conditions [43]. The degree of sulfation (DS) can be precisely controlled by varying the molar ratio of the sulfating reagent. The resulting product is typically purified by ethanol precipitation or dialysis followed by lyophilization [10].

Due to the acidic and mildly degradative nature of the synthetic environment, the average molecular weight of sHA ranges between 15 and 45 kDa, with a degree of sulfation between 1 and 3 mol. The primary hydroxyl group of the N-acetylglucosamine residue is preferentially sulfated because of its higher steric accessibility, whereas the remaining three hydroxyls undergo sulfation in a more random pattern (Figure 1).

Structurally, sHA closely resembles heparan sulfate, as both polymers are composed of glucuronic acid (GlcA) and N-acetylglucosamine (GlcNAc). However, they differ in glycosidic linkages, acetylation patterns, and sulfation positions [44,45,46]. Highly sulfated HAs (e.g., sHA3) share certain physicochemical properties with heparin, although they lack its specific 3-O-sulfation site, explaining their significantly reduced anticoagulant activity [45,46].

Indeed, kinetic chromogenic anti-Xa assays show that sHA1 lacks measurable anticoagulant activity, while sHA3 exhibits a mild effect—approximately thirty times weaker than heparin [47].

As a native extracellular matrix (ECM) component, HA is inherently biocompatible, and similar results have been confirmed for sHA in cytotoxicity assays on fibroblast cell lines (IC_50_ = 7.7 ± 0.7 mg/mL for sHA2) [47,48].

Chemical sulfation markedly enhances HA’s resistance to enzymatic degradation by hyaluronidase. Lemmnitzer et al. [49] demonstrated that sulfated HA substrates undergo significantly slower enzymatic depolymerization compared to native HA. Increasing the degree of sulfation corresponded to a progressive reduction in enzyme velocity for both human (PH20) and bovine (BTH) hyaluronidases. Moreover, specific inhibition assays revealed a direct correlation between inhibitory potency and sulfation degree [49], suggesting a longer in vivo residence time and improved therapeutic persistence of sHA formulations. To better illustrate these biochemical differences, the main structural and functional distinctions between native HA and sulfated HA are summarized in Table 3.

Beyond its stability, sHA exhibits several functional advantages relevant to tissue regeneration and inflammation [50,51,52,53]. Like native HA, it promotes fibroblast adhesion and spreading by binding fibronectin and preserving its conformational integrity [50,51,52]; The additional sulfate groups enable electrostatic interactions with locally or globally positively charged proteins—such as cytokines, chemokines, and growth factors—thereby modulating their local concentration, shielding them from proteolytic degradation, and facilitating receptor presentation [53,54].

Another notable feature of sHA is the presence of β(1→4) and β(1→3) glycosidic linkages between D-glucuronic acid and N-acetyl-D-glucosamine residues. This β configuration stabilizes the polysaccharide’s extended conformation, enhances its water-retaining capacity, and supports selective binding to protein ligands [20,55].

Such β-linkages, rare among natural sulfated GAGs, distinguish sHA from heparan sulfate and chondroitin sulfate—most of which possess α-linkages—and confer increased rigidity and molecular recognition capability [56,57,58]. This structural organization contributes to the exceptional hydration and substrate-binding properties of sHA, underpinning its potential biomedical applications in dermatology, wound repair, and regenerative medicine [20].

## 5. Rationale for Using Sulfated Hyaluronan in Dermatology

Although high-molecular-weight hyaluronic acid (HMW-HA) forms a protective film on the skin surface and strengthens the barrier by reducing transepidermal water loss, its large size limits epidermal penetration. In contrast, low-molecular-weight HA (LMW-HA) diffuses more efficiently into the epidermis [56,57], yet extremely low-mass fragments may elicit proinflammatory cytokine release and immune activation [58]. Sulfation of LMW-HA mitigates these undesired effects while enhancing its soothing and anti-inflammatory properties, making sulfated hyaluronic acid (sHA) an attractive active ingredient for topical dermatological applications [59].

sHA has been extensively investigated in both experimental and clinical models, particularly in wound healing and tissue regeneration [60]. For instance, sHA has been incorporated into hyaluronan/collagen composite hydrogels designed as carriers for heparin-binding epidermal growth factor (HB-EGF) [61]. This growth factor plays a key role in activating keratinocytes and fibroblasts during early wound repair. The high affinity of sHA for HB-EGF allows for controlled retention and gradual release, maintaining local bioactivity and creating a favorable microenvironment for keratinocyte migration, fibroblast proliferation, and accelerated re-epithelialization. Both in vitro and ex vivo skin models have demonstrated that sHA-enriched matrices promote superior epidermal regeneration compared with non-sulfated analogs [61].

In addition, Hintze et al. [62] and Koehler et al. [63] demonstrated through surface plasmon resonance (SPR) and computational analyses that sHA directly interferes with the Transforming Growth Factor-β1 (TGF-β1) signaling pathway by competitively binding to the cytokine and blocking its interaction with receptors TβRI and TβRII. The binding affinity increases proportionally to the sulfation degree, reaching its maximum with highly sulfated sHA_3_. This electrostatic interaction is mediated by the strong attraction between negatively charged sulfate groups and positively charged residues on the TGF-β1 surface. Consequently, sHA may locally reduce fibroblast activation and excessive collagen synthesis, representing a potential antifibrotic and remodeling agent for topical use in conditions associated with pathological TGF-β signaling, such as hypertrophic scars and sclerosing dermatoses.

Preclinically, sHA exhibits marked resistance to hyaluronidase degradation and enhanced retention of heparin-binding growth factors (VEGF, FGF, TGF-β) [7,13,49], leading to modulation of angiogenesis and inflammatory tone. In skin models, HA/collagen scaffolds enriched with sHA have shown improved dermal repair through attenuation of pro-inflammatory macrophage infiltration and promotion of epithelial closure [60,61]. These findings provide a biochemical and translational rationale for the use of sHA as both a co-excipient and active component in topical formulations targeting barrier restoration, wound healing, and inflammatory dermatoses (Table 4).

The unique biological and physicochemical properties of sHA, combined with its biocompatibility and safety profile, support its potential as a therapeutic or cosmeceutical agent in dermatology. Beyond wound healing, sHA demonstrates promising effects in reducing inflammation, enhancing skin hydration, and restoring homeostasis—critical factors in preventing relapse of chronic skin disorders [48].

### 5.1. Atopic Dermatitis

Atopic dermatitis (AD) is a chronic, inflammatory dermatologic condition affecting approximately 11–20% of children and 5–8% of adults, equating to over 230 million individuals worldwide [56]. It is characterized by pruritus, erythema, xerosis, and recurrent eczematous lesions, significantly impacting patients’ quality of life and mental health. AD exhibits clinical heterogeneity, ranging from mild, localized eczema to severe erythroderma, and often coexists with other atopic conditions, such as asthma and allergic rhinitis. Its pathogenesis involves a complex interplay of genetic, environmental, and immune factors, including filaggrin gene mutations, epidermal barrier dysfunction, Th2-polarized inflammation, and microbial dysbiosis, particularly with *Staphylococcus aureus* [57,58].

Oxidative Stress plays a significant role in worsening AD by promoting inflammation, damaging the skin barrier, and increasing allergen penetration [59], thus resulting in increased oxidative stress biomarkers in AD patients (e.g., urinary 8-hydroxydeoxyguanosine, malondialdehyde) and consequent symptoms onset as skin itching, dryness, lichenifications, and eczema-like skin lesions with erythematous patches and papules.

Overexpression of the enzyme HA-synthase (HAS3) in a model simulating atopic dermatitis (AD) leads to excessive HMW (HA) in the intercellular spaces between keratinocytes in the epidermis. This phenomenon is associated with spongiosis, a typical feature of AD skin, where reduced expression of epidermal cadherins facilitates water entry into the tissue. The presence of HMW HA in larger amounts than in normal skin and the reduction in cell junctions (cadherins) may favour HA accumulation and water retention in the enlarged intercellular spaces.

Thus, while low molecular weight fragments would contribute to hydration, HMW HA in excess in the cornified layer could counteract the formation of the normally hydrophobic cornified layer, impairing the skin’s barrier function [60].

Chronic inflammation and barrier dysfunction lead to decreased synthesis of sGAGs like HS and CS; the ECM becomes disorganized, impairing its ability to bind growth factors and maintain skin structure, and lower sGAG levels reduce the skin’s ability to retain moisture and regenerate, worsening the dryness and inflammation typical of AD [61]. Traditional management of AD focuses on avoiding triggers, on the regular use of emollients and on pharmacological treatments, including topical corticosteroids, calcineurin inhibitors, and systemic immunosuppressants for severe cases [62,63].

Novel therapies, such as biologics targeting IL-4, IL-13, or JAK pathways, have shown efficacy but are often limited by high costs and potential side effects [64]. Antioxidants such as Coenzyme Q10, vitamins and flavonoids are employed to reduce or neutralize the oxidative stress-driven effects.

The application of LMW HA or its derivatives has emerged as a promising adjunctive therapy for AD due to its multifaceted benefits [64,65]. Recent preclinical studies demonstrated that HA alleviates AD-like symptoms in mouse models, reducing epidermal thickening [66,67]. In addition, HA supports the wound healing process, indirectly favouring keratinocyte migration, proliferation, and extracellular matrix remodeling [68]. This is particularly beneficial in AD, where skin lesions are prone to irritation, infection, and delayed repair.

On the other hand, some sGAGs also find interesting applications as active ingredients in formulations useful for the treatment of AD. For example, polysulfated mucopolysaccharide (MPS), a heparinoid composed of polysulfated CS, is a moisturizing agent used for the treatment of xerosis in patients with AD.

MPS treatment significantly increased the mRNA and protein expression of claudin-1 (CLDN1) and zonula occludens-1, and significantly increased transepithelial electrical resistance (TEER), which indicates Tight Junction integrity. Conversely, CS and HA had little effect on TEER or the expression of mRNAs or TJ-related proteins [67,68,69]. By improving barrier integrity, MPS could serve as a novel therapeutic option for managing skin barrier dysfunction, particularly in conditions like atopic dermatitis, where tight junction defects are a known issue [33,55].

**Figure 2:**
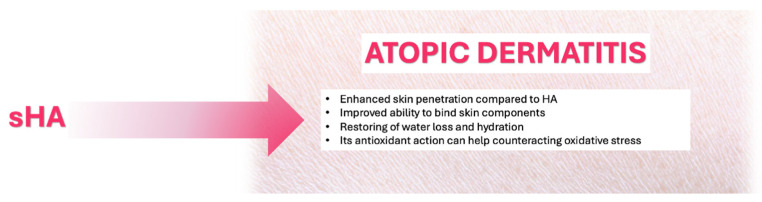
Proposed mechanisms of action of Sulfated Hyaluronic Acid (sHA) in Atopic Dermatitis.

A similar action to MPS can be hypothesized for sHA due to similar chemical structure; in vitro studies evidenced that sHA exhibits enhanced skin penetration compared to HA due to its lower molecular weight (regardless of the sulfation degree) and improved ability to bind skin components [70]; moreover, its high sulfation degree lead to high water binding capacity and can concur in restoring of water loss and hydration, while its antioxidant action [71] can help counteracting oxidative stress (Figure 2).

### 5.2. Psoriasis

Psoriasis is a chronic inflammatory skin disease with a relapsing-remitting course, characterized by erythematous plaques with a firm, whitish scale and a typical distribution pattern. It affects approximately 2% of the global population, with incidence peaking between the 3rd–4th and 6th–7th decades of life [72]. Psoriasis is multifactorial, involving a combination of genetic predisposition and environmental triggers, leading to a Th1-driven immune response and the release of key cytokines, including IL-17, IL-23, and TNF-α [73].

Psoriasis vulgaris accounts for the majority (80–90%) of cases, presenting with symmetrical erythematous-desquamative plaques primarily on the knees, elbows, lumbosacral area, and scalp. Other clinical forms include guttate psoriasis, erythrodermic psoriasis, and pustular psoriasis, each with distinct presentations and severity. Symptoms like pruritus, burning, and pain are common, particularly in fissured or flexural areas [74,75].

Management requires individualized therapy based on disease severity, clinical subtype, and patient quality of life. Options include topical therapies (corticosteroids, vitamin D3 analogs, retinoids, salicylic acid, and emollients), phototherapy (narrow-band UVB or UVA combined with psoralens), and/or systemic therapies, such as traditional options (methotrexate, cyclosporine, systemic retinoids) and biologics targeting TNF-α, IL-17, IL-12/23, or IL-23- [60,61,62]

Despite advancements, the need for safe, long-term management of mild to moderate forms persists. The use of sGAGs as an active ingredient in topical formulations for psoriasis treatment can find an interesting rationale in the restoration of the skin’s natural balance and in the reduction of the local adverse effects of long-term systemic therapies such as corticosteroids.

Priestley [76] reported an increased excretion of sGAGs in patients with psoriasis, suggesting an altered GAG metabolism in the disease. A pre-clinical study in a mouse model showed a correlation between the overexpression of heparinase (an endoglycosidase that selectively degrades HS) and the formation of psoriasis plaques [77], and the altered distribution of HS in psoriasis was also confirmed in a human histology assessment that shows a reduced GAG sulfation pattern in psoriasis patients [78].

Although these works do not directly establish a treatment effect, they lay biochemical groundwork that supports the potential use of sGAG supplementation as part of the therapeutic approach.

**Figure 3:**
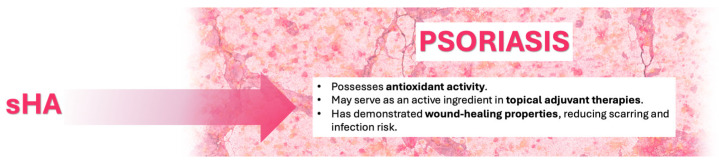
Proposed mechanisms of action of Sulfated Hyaluronic Acid (sHA) in Psoriasis.

Oxidative stress is well described as one of the major causes of psoriasis pathogenesis [44,79]. sHA polymers, thanks to their antioxidant activity [80], could be considered as active ingredients in a topical formulation for the adjuvant therapy of psoriasis; moreover, sHA has been used as a wound healing agent due to its ability to support the physiological healing process by reducing the risk of scarring and infection [81] (Figure 3).

### 5.3. Acne

Acne vulgaris is a multifactorial inflammatory disorder of the pilosebaceous unit, characterized by non-inflammatory (comedones) and inflammatory (papules, pustules, nodules) lesions. Its pathogenesis involves increased sebum production, follicular hyperkeratinization, colonization by *Cutibacterium acnes* (formerly *Propionibacterium acnes*) and *Staphylococcus* species, and activation of innate and adaptive immune responses [82,83]. Although acne predominantly affects adolescents, it may persist or newly arise in adulthood, particularly in women with hormonal imbalance such as polycystic ovary syndrome (PCOS) [84].

Conventional therapies—topical retinoids, benzoyl peroxide, antibiotics, and isotretinoin—are effective but limited by adverse effects (dryness, irritation, erythema, burning) and increasing antibiotic resistance [85]. Recently, hyaluronic acid (HA) has been investigated not only as a hydrating and regenerative molecule but also as a modulator of inflammation and sebaceous activity. Its capacity to restore skin barrier function, stimulate collagen synthesis, and enhance keratinocyte and fibroblast regeneration makes it a valuable adjunct in acne management [86].

In this context, the *C. acnes* interaction with HA and other glycosaminoglycans (GAGs) has gained attention. The bacterium expresses two distinct hyaluronate lyase (HYL) variants: HYL-IA, weakly active and mainly associated with acne-related surface strains, and HYL-IB/II, highly active and linked to deeper tissue invasion. These enzymes degrade HA within the extracellular matrix, potentially amplifying inflammation and tissue injury [87]. The degradation products of HA may further modulate host immune responses and favor bacterial colonization [88]. Therefore, exogenous supplementation with sulfated or modified HA derivatives might competitively inhibit bacterial adhesion to host GAGs, mitigating inflammation and scarring [89].

Moreover, innovative HA-based nanoconjugates, such as HA-FGF2-derived peptide bioconjugates (HA-P5), have shown potent anti-acne activity. In preclinical models, HA-P5 nanoparticles simultaneously suppressed fibroblast growth factor receptor-2 (FGFR2) and androgen receptor (AR) signaling in sebocytes, reduced sebum synthesis, and reversed acne-associated transcriptomic alterations. Unlike conventional FGFR inhibitors, HA-P5 avoided androgen-stimulating compensatory pathways. This dual modulation of FGFR2/AR signaling underscores the potential of HA derivatives as safe, biologically active agents for acne treatment [90,91,92].

**Figure 4:**
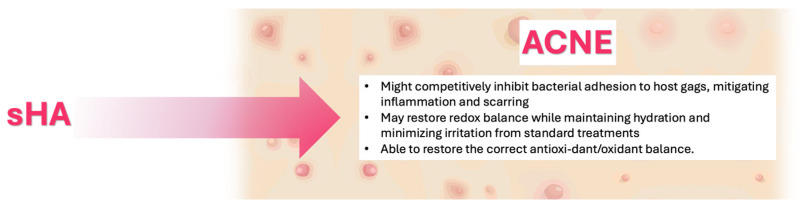
Proposed mechanisms of action of Sulfated Hyaluronic Acid (sHA) in Acne.

Finally, as observed in psoriasis and atopic dermatitis, oxidative stress contributes to acne pathogenesis [93]; thus, low-sulfated HA may also restore redox balance while maintaining hydration and minimizing irritation from standard treatments, and sHA administration could restore the correct antioxidant/oxidant balance (Figure 4).

### 5.4. Other Conditions

Beyond atopic dermatitis, psoriasis, and acne, the potential of sHA has also been explored in the context of wound healing and chronic skin lesions, such as pressure ulcers and diabetic wounds. In these conditions, impaired ECM remodeling, oxidative stress, and delayed re-epithelialization are major barriers to tissue repair. Preclinical studies demonstrated that sHA, by mimicking heparan sulfate, enhances fibroblast proliferation, angiogenesis, and collagen organization, while modulating the inflammatory response [7,60]. Its increased negative charge allows for high-affinity binding and prolonged retention of heparin-binding growth factors such as FGF2, VEGF, and HB-EGF, thus supporting controlled release and sustained bioactivity in the wound bed [61,62].

Analogous findings were reported for other HA-based derivatives, including FGF2-peptide bioconjugates, which improved dermal repair through enhanced growth factor stabilization and ECM reorganization (Hyaluronic acid–FGF2-derived peptide bioconjugates, 2023). These results collectively suggest that sulfation of HA confers biological advantages relevant to the management of chronic and hard-to-heal wounds, providing a favorable microenvironment for re-epithelialization and dermal regeneration.

For pressure ulcers, the same biochemical rationale applies: the combination of increased hydration, ECM stabilization, and growth-factor sequestration may promote tissue oxygenation and granulation. However, controlled clinical evidence remains scarce, and standardized evaluation of sHA formulations in this setting is still lacking.

Regarding impetigo and other superficial bacterial infections, no direct studies have yet examined the use of sHA. Nonetheless, the available literature suggests that pathogenic bacteria such as Staphylococcus aureus and Cutibacterium acnes adhere to host GAGs on keratinocyte surfaces as part of their colonization process. The introduction of sulfate groups into HA could, in theory, alter these electrostatic interactions and reduce bacterial adhesion, as previously observed for highly sulfated GAGs like heparin. Such anti-adhesive and anti-inflammatory potential warrants further investigation to assess whether sHA may help prevent microbial attachment and support the restoration of skin barrier integrity in infection-prone or wounded skin [90,94,95].

Overall, the application of sHA in wounds and infectious dermatoses remains a promising but underexplored area. Future research should focus on dose-response characterization, clinical standardization of sulfation degree, and evaluation in chronic wound and infection models to clarify its translational value in these indications.

## 6. Conclusions

Glycosaminoglycans (GAGs), both sulfated and non-sulfated, are key structural components of the extracellular matrix (ECM), essential for maintaining skin integrity, hydration, and repair [14,28,29]. Alterations in GAG expression or sulfation patterns occur during intrinsic and extrinsic aging and in inflammatory skin disorders such as psoriasis, atopic dermatitis, and acne [18,19,85,87,95]. These changes contribute to reduced water retention, impaired barrier function, and extracellular matrix disorganization, ultimately leading to clinical manifestations such as dryness, loss of elasticity, and wrinkle formation.

Among GAGs, hyaluronic acid (HA) plays a central role in cutaneous homeostasis, reducing transepidermal water loss, enhancing hydration, supporting wound repair, and counteracting oxidative stress [1,3,4,6]. However, the therapeutic use of native HA is limited by its rapid enzymatic degradation and short biological half-life.

Chemical sulfation of HA produces sulfated derivatives (sHA) with increased negative charge density, improving water retention, resistance to hyaluronidase degradation, and electrostatic interactions with growth factors and cytokines [7,41,42,43,44,45,46,47,48,49,50,52]. Preliminary evidence suggests that these modifications may enhance barrier repair, modulate inflammation, and promote extracellular matrix remodeling [60,61,63,80].

Nevertheless, this review is narrative in nature and may not encompass all available studies. Further investigations are warranted to standardize the degree of sulfation, evaluate potential dose-dependent effects, and conduct controlled clinical trials to confirm safety and efficacy in dermatological applications.

Although Sodium Sulfated Hyaluronate (INCI) is already commercially available as a cosmetic ingredient used for skin conditioning (COSMILE Europe Database, 2025 [96]), published clinical evidence on finished topical products remains limited. Continued exploration of sHA as both an active component and co-excipient in topical formulations may provide a complementary approach to conventional therapies for chronic inflammatory skin diseases.

In summary, while sulfated HA represents a promising evolution of native hyaluronic acid with potential applications in both cosmetic and medical dermatology, its clinical value awaits confirmation through rigorous translational and clinical studies.

## Figures and Tables

**Table 1:** Summary of structural and functional differences between HMW-HA and LMW-HA relevant to skin physiology.

Property/Function	HMW-HA	LMW-HA	References
Average molecular weight	>1000 kDa	50–500 kDa	[15,16,17,18,19,20]
Diffusion through epidermis	Low	High	[16,17]
Enzymatic degradation rate	Slow	Fast	[19]
Water-binding capacity	Very high	Moderate	[1,2,3,4,5,6]
Biological activity	Anti-inflammatory, space-filling	Pro-healing, pro-angiogenic	[9,21]
Receptor interaction (CD44, RHAMM)	Moderate	Stronger (higher mobility)	[14]
Effect on fibroblasts	ECM organization	Migration and proliferation	[21]
Role in wound healing	Structural support	Re-epithelialization and ECM remodeling	[3,9,21]
Typical applications	Fillers, moisturizers	Healing gels, regenerative formulations	[5,6]

**Table 3:** Comparative properties of native hyaluronic acid (HA) and sulfated hyaluronic acid (sHA).

Parameter	Native HA (Non-Sulfated)	Sulfated HA (sHA)
Stability to hyaluronidase	Low	High (slower enzymatic degradation)
Charge density	Negative (COO^−^)	More negative (COO^−^ + SO_3_^−^)
Growth factor binding (VEGF, FGF, TGF-β)	Limited	Increased (stronger sequestration and retention)
Receptor affinity (e.g., CD44)	Moderate	Modified or enhanced (depending on degree and pattern of sulfation)
Antioxidant capacity	Limited	Improved (preclinical evidence)
Skin penetration/absorption	Dependent on molecular weight	Influenced by both molecular weight and degree of sulfation; favorable electrostatic interactions
Dermatological implications	Hydration, barrier support	Barrier repair, anti-inflammatory effects, ECM remodeling

**Table 4:** Summary of preclinical and clinical studies evaluating sulfated hyaluronic acid (sHA) in dermatology.

Reference	Model/Method	System Studied	Main Findings	Relevance to Dermatology
[61]	Hydrogel delivery (in vitro/ex vivo)	Degradable hydrogels with sHA macromers	Sustained release of heparin-binding growth factors (e.g., HB-EGF); improved retention vs. non-sulfated HA	Controlled growth factor delivery and prolonged wound-healing response
[60]	Human outer-root-sheath keratinocytes and melanocytes (in vitro)	Artificial ECM containing sHA	↑ Keratinocyte proliferation and migration; support of melanocytic phenotype; excellent cytocompatibility	Cutaneous regeneration and re-epithelialization
[63]	Surface plasmon resonance (SPR) and computational modeling	sHA vs. chondroitin sulfate derivatives interacting with TGF-β1	sHA interferes with TGF-β1:TβRI/TβRII complex formation; sulfation-dependent binding	Mechanistic basis for anti-fibrotic and anti-scarring potential
[62]	SPR and molecular modeling	sHA derivatives with TGF-β1 and its receptors	Modulation of TGF-β1 signaling and receptor binding in a sulfation-dependent manner	Regulation of fibroblast activation and ECM remodeling
[64]	Human macrophages (in vitro)	Inflammatory signaling assays	sHA attenuates NF-κB activation and induces antioxidant response (HO-1, SOD)	Anti-inflammatory and antioxidative potential in inflamed skin
[48]	Mouse model (in vivo)	High-sulfated HA administration post-radiation	Amelioration of radiation-induced tissue injury without systemic anticoagulation	Demonstrates in vivo safety and cytoprotective effects of sHA

↑ denotes an increase in the listed biological processes.

## Data Availability

Not applicable. No new data were created or analyzed in this study. Data sharing is not applicable to this article.
